# Decreased Serum Brain-Derived Neurotrophic Factor in Poststroke Depression: A Systematic Review and Meta-Analysis

**DOI:** 10.3389/fpsyt.2022.876557

**Published:** 2022-05-19

**Authors:** Chunhui Zhang, Xuefang Wang, Qinghua Zhu, Yongxia Mei, Zhenxiang Zhang, Hui Xu

**Affiliations:** School of Nursing and Health, Zhengzhou University, Zhengzhou, China

**Keywords:** brain-derived neurotrophic factor, meta-analysis, poststroke depression, depression, systematic review

## Abstract

**Backgrounds:**

There were conflicting results on the comparison of brain-derived neurotrophic factor (BDNF) levels between poststroke depression (PSD) patients and stroke patients without PSD among previous studies. Thus, we conducted this systemic review and meta-analysis to explore the alteration of serum BDNF levels in PSD.

**Methods:**

This study included articles from the Web of Science and PubMed databases that were published before December 2021. STATA 12.0 software was used to compute the standardized mean difference (SMD) and 95% confidence interval (CI) regarding the comparison of serum BDNF in PSD and stroke patients without PSD.

**Results:**

We collected the mean value and standard deviation (SD) of serum BDNF in PSD and stroke patients without PSD from six studies (PSD: *n* = 268, stroke patients without PSD: *n* = 425). The present meta-analysis showed decreased serum BDNF level in patients with PSD, compared to stroke patients without PSD with a random-effects model (mean value of BDNF level [PSD vs. stroke patients without PSD]: 14.106 vs. 17.995 ng/ml; SMD = –1.578; 95% CI: –2.820, –0.337; *I*^2^ = 97.8%, *p*-value for Q test < 0.001).

**Conclusion:**

Brain-derived neurotrophic factor may work as a potential biomarker to predict the risk of PSD among stroke survivors. More large-sample clinical trials exploring the alteration of serum BDNF levels in PSD among stroke patients need to be conducted to verify this result.

## Introduction

Poststroke depression (PSD), as an important complication of stroke, is very common accounting for 30–35% of patients following a stroke (1). Early-onset PSD that usually occurs within the first 3 months after stroke is more frequent than late-onset PSD (2). PSD is significantly associated with poor functional outcomes, affecting the life quality of patients and the rehabilitation of motor and cognitive deficits after stroke (3, 4). Worryingly, PSD significantly increases the risk of mortality in stroke survivors, and the hazard ratio for PSD and all-cause mortality was 1.59 (95% CI: 1.30–1.96) according to a meta-analysis (5). The pathogenesis of PSD is complex and multifactorial involving biological and social psychological mechanisms (6). Biological mechanisms included monoamine neurotransmitter change (7), inflammation mechanism (8), hypothalamus-pituitary-adrenal axis, and hypothalamus-pituitary-thyroid axis hypothesis (9). The onset of PSD is not a single mechanism. Whyte and Mulsant (7) put forward that PSD is under the bio-psycho-social medical model, and both biological factors and psychological factors could cause the onset of PSD. Studies reported that educational level (10), age (11), gender (12), preexisting personality characteristics of the patient (13), the marital status of the patient, the severity of stroke (14), chronic diseases (15), and social support (16) are correlated with the occurrence of PSD. Besides PSD is often associated with symptoms of cognitive impairment such as agnosia and memory changes, which may be mistakenly regarded as the consequences of stroke or old age, so it was estimated that approximately 50–80% of PSD cases were underdiagnosed (2, 17). PSD creates a heavy burden to individuals, families, and society.

Brain-derived neurotrophic factor (BDNF) promotes the survival of neurons by regulating nerve function, promoting nerve growth, increasing synaptic plasticity, and delivering efficiency (18). It could prevent the pathological changes of stroke, inhibit the apoptosis of neurons and effectively improve the neural function of stroke patients (6). BDNF is a secretory growth factor that mainly contributes to the regeneration of nerve and the development and plasticity of the nervous system *via* tyrosine kinase receptor B (TrkB) (19). BDNF, which is mainly secreted by the central nervous system, and its mRNA and TrkB are expressed in the hypothalamus, limbic system, and other areas of the brain (20). Past studies showed that in the socially stressed mice model BDNF levels were increased and BDNF-TrkB signaling contributed to depressive-like outcomes (21, 22). More and more evidence revealed that BDNF plays a major role in a number of mental diseases including depression, schizophrenia, and Alzheimer’s disease (AD) (23). Although BDNF has been one of the widely studied neurotrophins and suggested as a potential biomarker of brain pathological conditions, we still lack an understanding of the mechanisms that result in the pathological changes of BDNF. Such neurotrophic factors regulate nerve function, promote nerve growth, and increase synaptic plasticity and delivery efficiency (24). A neurotrophic factor is a molecule that promotes the development and survival of neurons. It can prevent the pathological changes of ischemic brain injury, and it can reduce the apoptosis of neurons and effectively improve the neurological function of patients after stroke.

However, there were conflicting results in comparison of BDNF levels between patients with PSD and stroke patients without PSD among previous studies. Zhou et al. (25) reported that the levels of BDNF in patients with PSD were higher than in stroke survivors without depression, while several studies showed the opposite result (26, 27). Thus we conducted this systemic review and meta-analysis to explore the alteration of serum BDNF level in PSD development and evaluate whether BDNF level is an applicable biomarker candidate for predicting the risk of PSD development at the early stage of stroke.

## Materials and Methods

This study was conducted on the basis of the Preferred Reporting Items for Systematic Reviews and Meta-Analyses (PRISMA) statement (28).

### Search Strategy and Selection Criteria

This study included articles published before December 2021 and searched in Web of Science and PubMed databases. Included studies should be in English. We used search terms as follows: (“brain-derived neurotrophic factor” OR “BDNF”) AND (“poststroke depression” OR “PSD”). After removing duplicates, 345 studies explored the alteration of serum BDNF levels in PSD. Studies did not provide sufficient information about the alteration of serum BDNF levels in PSD. In addition, reviews, meta-analysis, and case reports were excluded.

### Data Collection and Meta-Analysis

Two researchers read the titles and abstracts of all articles. We collected the following data from included articles: Author and publication years, study location, sample size, gender, mean age, serum BDNF concentrations, time interval, PSD diagnostic criteria, follow-up duration, sample type, and detection method. STATA version 12.0 software was used to compute standard mean difference (SMD) and 95% confidence interval (CI) regarding the comparison of serum BDNF in PSD and stroke patients without PSD. Heterogeneity between studies was assessed with I^2^ and Q tests. When the heterogeneity was high (*I*^2^ ≥ 50%, *p-*value for Q test ≤ 0.05), we used random-effects models to compute results, performed as pooling methods. Inversely, fixed-effects models were used as pooling methods. Meta-regression analysis was applied to explore the source of heterogeneity. Sensitivity analysis was used to evaluate the stabilization of meta-analysis. Finally, Begg’s test, Egger’s test, and funnel plot were used to evaluate publication bias. Quality appraisal was made using the Cochrane Risk of Bias Tool. Data were analyzed using Review Manager version 5.3.

## Results

### Search Results

**FIGURE 1:**
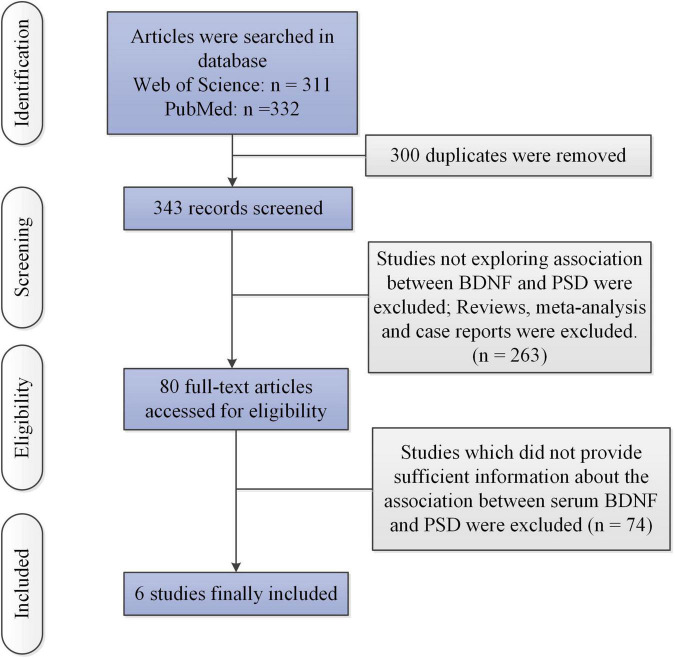
The flow of information through the different phases of a meta-analysis.

Figure 1 illustrates the selection process. Table 1 reports important elements of finally included six articles. We collected the mean value and standard deviation (SD) of serum BDNF in PSD and stroke patients without PSD from six studies (24–27, 29, 30) (PSD: *n* = 268, stroke patients without PSD: *n* = 425).

**TABLE 1:** Study characteristics of included studies.

References	Country	Sample size	Gender (Male %)	Age	BDNF (ng/mL)*	Stroke type	Time interval	PSD diagnosis	Follow-up duration	Sample type	Detection method
Yang et al. (29)	China	PSD: 37	48.6%	69.0 (9.3)	5.26 (1.05)	Ischemic stroke	<24 h	DSM-IV	14 days	Serum	ELISA
		Non-PSD: 63	58.7%	68.4 (11.2)	6.62 (1.18)						
Zhou et al. (25)	China	PSD: 35	54.3%	61.7 (8.5)	29.1 (11.4)	Ischemic stroke	<72 h	DSM-IV	6 months	Serum	ELISA
		Non-PSD: 58	58.6%	63.5 (12.5)	28.1 (9.7)						
Li et al. (24)	China	PSD: 59	40.7%	72.8 (11.2)	8.1 (5.6-9.4)	Ischemic stroke	<24 h	DSM-III-R	3 months	Serum	ELISA
		Non-PSD: 157	59.2%	63.6 (9.1)	13.7 (10.4-16.5)						
Li et al. (30)	China	PSD: 40	45%	59.3 (9.1)	16.754 (4.451)	Ischemic stroke	8–72 h	HRSD	6 months	Serum	ELISA
		Non-PSD: 50	52%	61.2 (11.2)	29.551 (3.213)						
Syafrita et al. (26)	Indonesia	PSD: 36	50%	59.7 (9.7)	6.44 (1.75)	Ischemic stroke	<48 h	HRSD	1 month	Serum	ELISA
		Non-PSD: 36	52.8%	59.6 (11.2)	7.52 (1.64)						
Han et al. (27)	China	PSD: 61	57.4%	67.9 (13.2)	18.97 (6.26)	Ischemic or Hemorrhagic Stroke	NA	DSM-IV	NA	Serum	ELISA
		Non-PSD: 61	60.7%	68.0 (16.8)	22.48 (7.68)						

*Values were expressed as mean (SD) or medians (IQR). Time interval between stroke onset and hospital admission. BDNF, brain-derived neurotrophic factor; ELISA, enzyme-linked immunosorbent assay; IQR, interquartile range; PSD, poststroke depression; SD, standard deviation.

### Meta-Analysis Results

**FIGURE 2:**
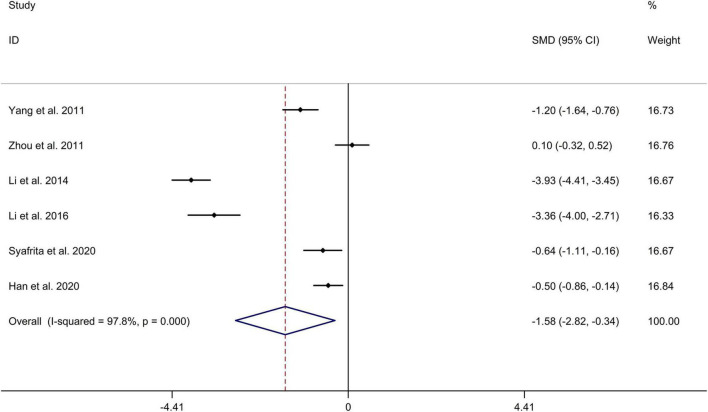
Forest plots of comparison of serum BDNF level between patients with PSD and stroke patients without PSD. BDNF, brain-derived neurotrophic factor; PSD, poststroke depression.

**FIGURE 3:**
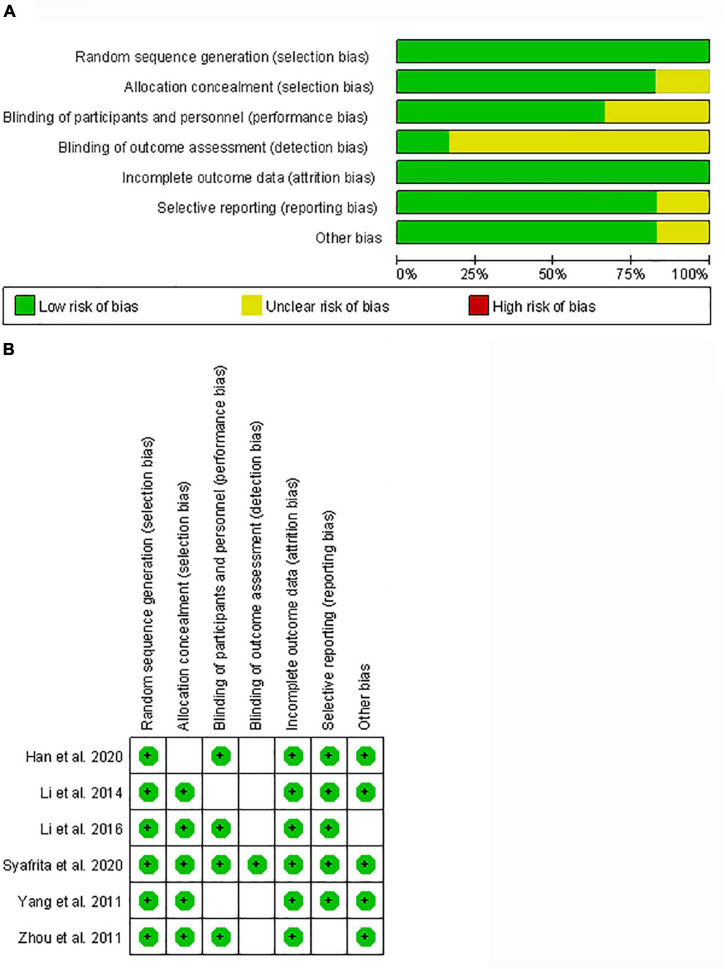
Risk of bias graph **(A)** and risk of bias summary **(B)**.

This meta-analysis showed decreased serum BDNF level in patients with PSD, compared to stroke patients without PSD with a random-effects model (mean value of BDNF level [PSD vs. stroke patients without PSD]: 14.106 vs. 17.995 ng/ml; SMD = –1.578; 95% CI: –2.820, –0.337; *I*^2^ = 97.8%, *p-*value for Q test < 0.001; Figure 2). Meta-regression analysis indicated that gender and age were not responsible for heterogeneity across studies (gender: *p-*value = 0.056; age: *p-*value = 0.559). Sensitivity analysis indicated no changes in the direction of effect when any one study was excluded, which showed the stability of the results (Supplementary Figure 1). In addition, Begg’s test, Egger’s tests, and funnel plots indicated no significant risk of publication bias (Begg’s test: *p* = 0.133; Egger’s test: *p* = 0.165; Supplementary Figure 2). The risk of bias graph and details of the risk of bias summary are shown in Figure 3.

## Discussion

In this systemic review and meta-analysis, our findings showed that compared to patients without PSD, patients with PSD had lower levels of BDNF (SMD = –1.578, 95% CI: –2.820, –0.337). This result may suggest that BDNF is a potential biomarker for predicting the risk of PSD development at the acute stage of stroke. A previous meta-analysis indicated that patients with stroke who had significantly lower serum BDNF concentrations at the early stage of stroke were associated with a higher risk of developing PSD (SMD = –1.43, 95% CI: –2.56, –0.31), which was consistent with our result (31). BDNF plays an important role in functional neurological rehabilitation after stroke and reduces the risk of stroke (32). Low serum BDNF has been found in patients with vascular risk factors related to cerebrovascular disease (33). Some studies (25, 34) showed a BDNF time curve after stroke. That study (25) reported that no significant differences in serum concentrations of BDNF were shown between patients with PSD and without PSD in the acute stage of stroke. For patients without PSD, insignificant changes were shown in BDNF between 7 days and 6 months after the stroke. A previous study reported that serum BDNF levels did not differ between the patients with and without PSD at 1 month after stroke (34). Another study found decreased serum BDNF concentrations in patients with PSD at 3–6 months after stroke (25).

Although our systemic review and meta-analysis came up with a meaningful result, we should notice the differences between included studies. First, Han et al. (27) included both patients with ischemic stroke and hemorrhagic stroke, while other studies only included patients with ischemic stroke. Second, the follow-up durations of included studies were different, varying from 14 days to 6 months. Third, the time intervals between stroke onset and hospital admission among included studies were also different, ranging from 8 to 72 h. These differences in the above factors may affect the accuracy of our findings.

Furthermore, the exact mechanisms involved in the changes between BDNF levels and PSD development remain to be further studied. It is widely assumed that in certain brain disorders, the chronic inflammatory state leads to an abnormal change in BDNF levels, because several BDNF-related signaling pathways can be affected by nerve inflammation (35). There were evidences that lipopolysaccharide (LPS) can induce a depression-like phenotype by inflammation in the prefrontal cortex, hippocampus, and nucleus accumbens *via* BDNF-TrkB signaling, and antibiotic drug minocycline can block LPS-induced sickness and depression-like behaviors in mice models (36). Studies also revealed that the change of BDNF did not happen in every area of the whole brain. Yang et al. (37) reported that lower levels of mature BDNF in the parietal cortex in postmortem brain from major depressive disorder (MDD), schizophrenia (SZ), and bipolar disorder (BD) groups were detected by western blotting analysis, compared to the control group, whereas no significant difference in levels of BDNF in the cerebellum in postmortem brain between MDD, SZ, and BD groups and controls. In addition, the increased levels of BDNF were shown in the livers of MDD, SZ, and BD groups, compared to the control group. Moreover, a negative correlation was found between mature BDNF in the parietal cortex and mature BDNF in the liver in all the subjects. The results support that production abnormality of mature BDNF in the brain and liver might play a role in the pathophysiology of psychiatric disorders, demonstrating a brain-liver axis in psychiatric disorders. In humans, decreased BDNF levels were found in the anterior cingulate cortex and caudal brainstem of suicide decedents compared with non-suicide decedents (38). Maheu et al. (39) reported that there were no differences in BDNF levels between healthy controls and individuals with a history of depression. Misztak et al. (40) showed that compared to controls the expression of BDNF was significantly reduced in the frontal cortex and hippocampus of suicide victims.

There are some limitations to our study. First, in addition to being very few included studies, most of the studies included are from China. All included studies were conducted in Asia, and the study population was yellow race. Second, studies indicated that the change of BDNF may differ in serum and brain, thus it is still unclear whether a variation of serum BDNF can accurately reflect the change of BDNF in the brain (35). Third, included articles used commercially available BDNF enzyme-linked immunosorbent assay (ELISA) kit got the measurement of serum BDNF. It is well-recognized that these ELISA kits can recognize mature BDNF and its precursor pro-BDNF because of the lack of specificity of antibodies (41, 42). Therefore, the values of BDNF using these ELISA kits are total values of mature BDNF and pro-BDNF in human serum. Fourth, the articles included in the study were not comprehensive because we could not obtain the article except in English and Chinese.

In conclusion, in this meta-analysis, we showed that BDNF may work as a potential biomarker to predict the risk of PSD among stroke survivors. More large sample clinical trials exploring the alteration of serum BDNF levels in PSD among stroke patients are needed to be conducted to verify this result.

## Data Availability Statement

The original contributions presented in the study are included in the article/Supplementary Material, further inquiries can be directed to the corresponding author.

## Author Contributions

CZ: manuscript writing, study search, and data collection. XW: study search, data collection, and data analysis. QZ and YM: data analysis. ZZ: data collection. HX: manuscript writing and revision, supervision, and funding. All authors contributed to the article and approved the submitted version.

## Conflict of Interest

The authors declare that the research was conducted in the absence of any commercial or financial relationships that could be construed as a potential conflict of interest.

## Publisher’s Note

All claims expressed in this article are solely those of the authors and do not necessarily represent those of their affiliated organizations, or those of the publisher, the editors and the reviewers. Any product that may be evaluated in this article, or claim that may be made by its manufacturer, is not guaranteed or endorsed by the publisher.
