# Supplementary material for: Decreased Serum Brain-Derived Neurotrophic Factor in Poststroke Depression: A Systematic Review and Meta-Analysis
Source: Front Psychiatry. 2022 May 19;13:876557. doi: 10.3389/fpsyt.2022.876557 (PMC9160429; doi:10.3389/fpsyt.2022.876557)
Supplement: Supplementary file 2 [file Image_1.pdf]

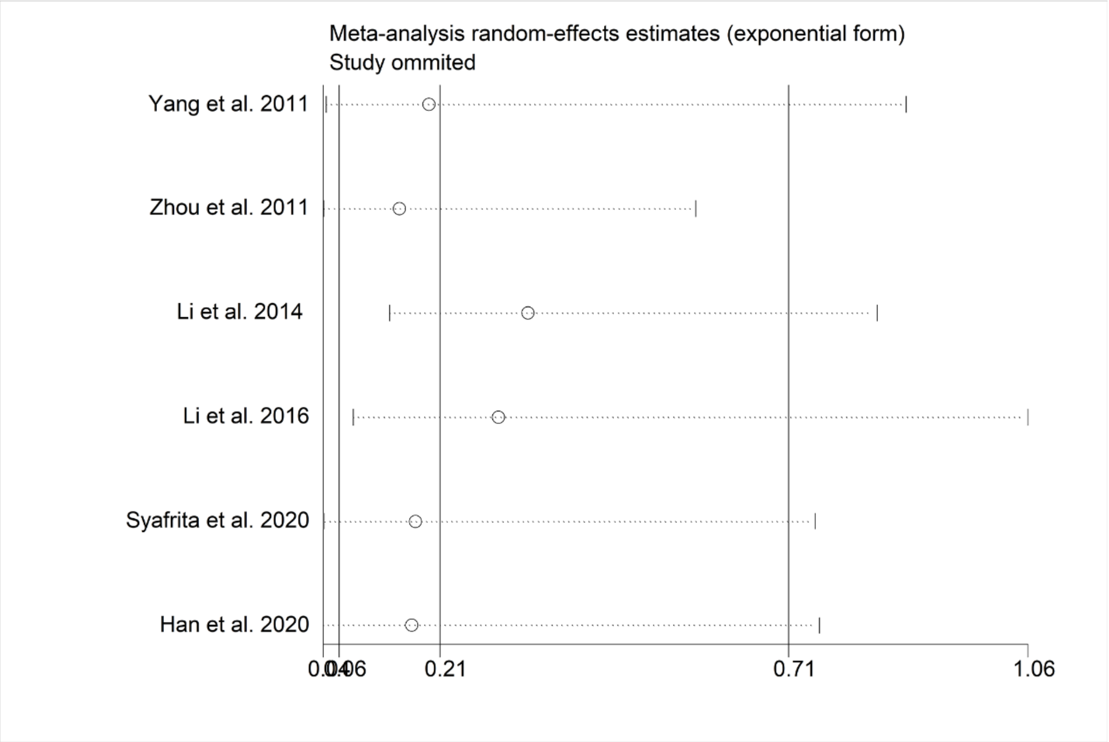

Supplementary figure 1. Sensitivity analysis regarding comparison of serum BDNF level between PSD patients and stroke patients with no PSD. Abbreviations: BDNF, brain-derived neurotrophic factor; PSD, post-stroke depression.
